# Epidemiology of hepatitis C virus infection & liver disease among injection drug users (IDUs) in Chennai, India

**Published:** 2010-12

**Authors:** Shruti H. Mehta, Samantha L. Vogt, Aylur K. Srikrishnan, Conjeevaram K. Vasudevan, Kalilapuri G. Murugavel, Shanmugam Saravanan, Santhanam Anand, M. Suresh Kumar, Stuart C. Ray, David D. Celentano, Suniti Solomon, Sunil S. Solomon

**Affiliations:** * *Department of Epidemiology, Johns Hopkins Bloomberg School of Public Health, Baltimore. USA*; ** *YR Gaitonde Centre for AIDS Research & Education (YRGCARE), Chennai, India*

**Keywords:** APRI, HCV genotype, hepatitis C virus, HIV, injection drug users, liver disease

## Abstract

**Background & Objectives:**

We characterized HCV antibody prevalence, viral persistence, genotype and liver disease prevalence among IDUs in Chennai, India as the study of the association of HIV with each of these states is important and there are no data available.

**Methods:**

Between 2005-2006, 1158 IDUs were recruited and followed semi-annually. All were tested for HCV antibodies at baseline; a random sample of 400 antibody positives (200 HIV-positive and 200 HIV-negative) were tested for HCV RNA; 13 of these were sequenced. Assessment of asparate amino transferase (AST)-to-platelet ratio index (APRI) was done on 557 IDUs. Prevalence ratios of each outcome were examined.

**Results:**

Median age was 35 yr; 99 per cent were male. HCV antibody prevalence was 55 per cent and was associated with older age, being unmarried, longer injection history, tattoo and injecting at a dealer’s place. Of the 400 HCV antibody positive IDUs, 281 (70.3%) had persistent infection which was less common among hepatitis B-infected persons but not associated with HIV. Of the 13 samples sequenced, 11 (85%) were HCV genotype 3a. Fibrosis prevalence according to APRI was: HIV/HCV-uninfected, 4 per cent; HIV mono-infected, 3 per cent; HCV mono-infected, 11 per cent; HIV/HCV co-infected, 12 per cent (*P*<0.001). In addition to being associated with HCV and HIV/HCV, fibrosis prevalence was higher among those drinking alcohol frequently; daily marijuana use was protective.

**Interpretation & Conclusions:**

Our findings show that IDUs in Chennai have high HCV prevalence and associated disease burden. The burden will increase as access to antiretroviral therapy improves particularly given the high prevalence of HIV, HCV and alcohol use.

Injection drug users (IDUs) are at high risk for blood-borne infections, including hepatitis C virus (HCV). HCV prevalence among IDUs in most countries has been reported to be at least 50 per cent with some estimates exceeding 90 percent^1^. HCV can lead to chronic liver disease causing cirrhosis, hepatocellular carcinoma and end-stage liver disease among 5-20 per cent of infected persons^2^. HCV-associated liver disease progression tends to be accelerated among individuals who are older, drink alcohol and are co-infected with HIV^3^,^4^. In fact, liver disease among HIV+ IDUs in the developed world has become an important cause of morbidity and mortality in the era of highly active antiretroviral therapy (HAART) as HIV-infected persons are living longer^5^. Less is known about the natural history of HCV among IDUs in developing countries, but as access to HAART improves in these countries, a similar pattern of HCV-related morbidity is expected.

India is estimated to have 168,000 - 1.1 million IDUs^6^,^7^ with HCV antibody prevalence ranging from 5 to 93 percent^8^–^10^; among HIV+ IDUs it is as high as 100 per cent^11^. Little data exist beyond antibody prevalence - a marker of exposure to HCV - among IDUs in India. Characterization of viral persistence and liver disease burden is important because co-factors which may impact HCV pathogenesis are different in India (*e.g*., heavy alcohol use, high prevalence of infections such as tuberculosis, HIV subtype C and poor nutritional status). We characterized prevalence and correlates of exposure to HCV, HCV viral persistence, liver disease and circulating HCV genotypes among a community-based cohort of IDUs in Chennai, India.

## Material & Methods

### 

#### Study population:

A prospective cohort study of IDUs [The Madras Injection Drug User and AIDS Cohort Study (MIDACS)] was initiated in Chennai, India in 2005 through YR Gaitonde Centre for Substance Abuse-Related Research (YRGCSAR). The primary objective of MIDACS was to characterize HIV incidence and the natural history of injection drug use among IDUs in Chennai. A convenience sample of 1158 IDUs was recruited via field staff who were acquainted with IDUs and their injection venues in Chennai as previously described^12^. To be included, participants had to be ≥ 18 yr, provide written informed consent and report a history of injection drug use in the prior six months. HIV negative participants were invited to return for semi-annual follow up visits and HIV+ participants were referred to an on-site HIV clinic where visits were structured based on clinical status. The study was approved by the Johns Hopkins Bloomberg School of Public Health and the YR Gaitonde Centre for AIDS Research and Education (YRGCARE) Institutional Review Boards.

#### Measurements:

At baseline, all study participants (n=1158) were tested for HCV antibodies using the Abbott Murex Anti-HCV kit, version 4.0 (Murex Biotech Limited, Kyalami, Republic of South Africa). Among HCV antibody positives at baseline, a stratified random sample (by HIV status) was selected for HCV RNA quantification (n=200 HIV+ and 200 HIV-). This sample size was selected to ensure sufficient statistical power to detect differences in the frequency of viral persistence by HIV serostatus. HCV RNA testing was performed using the COBAS Amplicor Monitor Version 2.0 (Roche Molecular Diagnostics, Branchburg, USA). The lower limit of detection was 600 IU/ml. A random sample of 50 persons with detectable HCV RNA was selected for HCV genotype testing. The testing was performed in batches of 10 sequentially until each subtype detected in the first 10 was represented at least twice for a total of 13 samples. HCV genotype testing was performed according to the Core/E1 RT-PCR protocol using the CE1_ISP primer as previously described^13^. At the 30-month follow up visit, a liver panel and complete blood count were performed in real-time among all participants who returned for the visit irrespective of baseline HIV and/or HCV status (n=557).

At baseline, HIV serostatus was determined using double enzyme-linked immunosorbent assay testing (Abbott Murex HIV-1.2.0; Murex Biotech Limited, Dartford, United Kingdom, and Vironostika HIV Uniform II Ag/Ab, BioMerieux, Boxtel, The Netherlands), and chronic HBV infection was determined by the presence of hepatitis B surface antigen (HBsAg; Hepanostika HBsAg Uniform II, BioMerieux, The Netherlands).

All study participants completed a questionnaire at baseline and semi-annual follow up visits that was administered by trained interviewers. At baseline, participants provided information on general demographics and answered questions pertaining to lifetime risk behaviours and risk in the prior one month. At follow up visits, questions were only asked about behaviours in the prior one month. Questions captured information on living conditions, sexual risk behaviour, drug use (injection, non-injection and alcohol) and drug-related risk behaviours.

#### Statistical analysis:

Chi-squared and Mann-Whitney tests were used to compare characteristics of HCV antibody positive and negative individuals. Univariate and multivariate Poisson regressions with robust variance estimates were used to calculate prevalence ratios of HCV antibody status by covariates of interest^14^. For this analysis, only demographic and lifetime history variables were considered to ensure temporality of exposures and outcome. HCV persistence (chronic infection) was defined by presence of detectable HCV RNA. The prevalence of significant liver disease was ascertained by calculating the aspartate aminotransferase (AST) to platelet count ratio index (APRI) on the 557 individuals who returned for their 30 month visit (APRI = AST (/ULN)/ Platelet count (10^9^/l) × 100)^15^. Validated cut-offs were used to estimate prevalence of significant liver disease. As previously defined, APRI >1.5 designated significant fibrosis, 0.5-1.5 designated mild/moderate fibrosis and APRI <0.5 designated no fibrosis^15^. Correlates of HCV persistence and significant liver disease were also calculated using Poisson regression with robust variance estimates. For the analysis of HCV persistence, in addition to lifetime variables, recent risk behaviours were considered given that HCV RNA positivity is dynamic as IDUs acquire and clear multiple infections^16^. In the analysis of liver fibrosis, exposure to alcohol, marijuana, buprenorphine and other pharmaceuticals were assessed at each visit and longitudinal exposure variables for each substance were created to represent the percentage of visits where an individual reported history of recent use of each particular substance. For all multivariate models, variables were considered if statistically significant at the *P*<0.10 level in univariate analysis or if determined *a priori* to be important. All statistical analyses were performed using Intercooled STATA version 10.1 (College Station, Texas).

## Results

### 

#### Characteristics of study population:

All but 3 of the 1,158 IDUs were male; 98.6 per cent were Tamil and the median age was 35 yr (Table I). The majority had less than a secondary school education, worked for daily wages and had income less than USD 72 per month. The median age at initiation of drug use was 25 yr [interquartile range (IQR): 20-30]. The majority consumed alcohol at least once per week. Among those who reported alcohol use, the median number of drinks per drinking episode was 4 (IQR: 4-4).

**Table I:** Characteristics of 1158 IDUs in the Madras Injection Drug User and AIDS Cohort Study, Chennai, India (2005-2009)^a^

	Total Cohort (n = 1158)	HCV negative (n = 527)	HCV positive (n = 631)	*P* value
Median age (IQR) yr	35 (29 - 40)	34 (27 - 41)	35(31 - 39)	<0.01
Ethnicity				0.21
Tamil	1,142 (98.6)	523 (99.2)	619 (98.1)	
Other	16 (1.39)	4 (0.76)	12 (1.90)	
Marital status				<0.001
Single	356 (30.7)	136 (25.8)	220 (34.9)	
Married/living with partner	764 (64.4)	376 (71.4)	369 (58.4)	
Separated	41 (3.5)	9 (1.7)	32 (5.1)	
Divorced/widowed	16 (1.36)	6 (1.14)	9 (1.56)	
Educational attainment				0.06
None	323 (27.9)	158 (30.0)	165 (26.2)	
Primary	393 (33.9)	189 (35.9)	204 (32.3)	
Secondary	295 (25.5)	126 (23.9)	169 (26.8)	
High school/university/professional	147 (12.7)	54 (10.3)	93 (14.7)	
Type of employment				<0.001
Monthly wages	75 (6.5)	26 (4.9)	49 (7.8)	
Weekly wages	61 (5.3)	20 (3.8)	41 (6.5)	
Daily wages	910 (78.6)	444 (84.3)	466 (73.9)	
Unemployment	112 (9.7)	37 (7.0)	75 (11.9)	
Monthly income				<0.001
<USD 12	119 (10.3)	41 (7.8)	78 (12.4)	
USD 12-36	499 (43.1)	249 (47.3)	250 (39.6)	
USD 37-72	437 (37.7)	207 (39.3)	230 (36.5)	
>USD 72	103 (8.9)	30 (5.7)	73 (11.6)	
Frequency of alcohol consumption				<0.001
Never	244 (21.1)	82 (15.6)	162 (25.7)	
Less than once a week	372 (32.1)	140 (26.6)	232 (36.8)	
More than once or once per week	542 (46.8)	305 (57.8)	237 (37.6)	
Median age at initiation of drug use (IQR) yr	25 (20-30)	25 (20-32)	23 (20-28)	<0.001
Frequency of drug injection^b^				<0.001
None	281 (24.3)	162 (30.7)	119 (18.9)	
< daily	688 (59.4)	335 (63.6)	353 (55.9)	
≥ daily	189 (16.3)	30 (5.7)	159 (25.2)	
Drugs used by injection^b^				
Heroin	609 (52.6)	201 (38.1)	408 (64.7)	
Buprenorphine	175 (15.1)	124 (23.5)	51 (29.1)	
Both	93 (8.0)	50 (7.6)	53 (8.4)	
Daily marijuana use^b^	473 (40.8)	212 (40.2)	260 (41.2)	0.74
Any pharmaceutical drug use^b^	399 (34.5)	139 (26.4)	269 (41.2)	<0.001
HIV positive	293 (25.3)	16 (3.0)	277 (43.9)	<0.001
Chronic HBV infection	119 (10.3)	55 (10.4)	64 (10.1)	0.87

a Data are presented as N (%) unless otherwise specified and *P* values were calculated using χ^2^ test or the Mann-Whitney test. IDUs, injection drug users; HCV, hepatitis C virus; HBV, hepatitis B virus; IQR, interquartile range;

b refers to behaviours in the prior one month at baseline. Missing data for drugs used by infection reflect individuals who did not inject in the prior one month at baseline

**Fig. 1:**
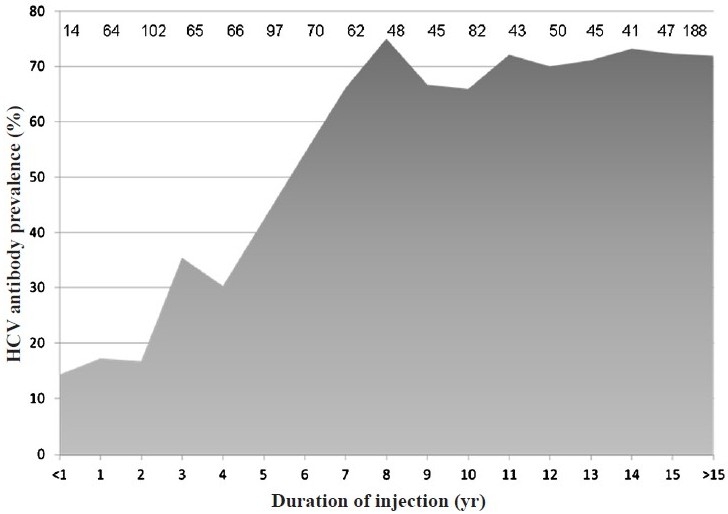
HCV antibody prevalence by years of injection drug use. Numbers of individuals contributing to the calculation of prevalence for each category of years of injection drug use are listed.

Overall, 631 (55%) were HCV antibody positive. The prevalence among recent initiates into injection was fairly low (14% among those injecting for <1 yr), but increased to 35 per cent for those injecting for 3 yr and peaked at 75 per cent for those injecting >8 yr (Fig. 1). Compared with HCV negative individuals, at baseline, HCV+ IDUs were more likely to inject daily, inject heroin vs. buprenorphine, abuse pharmaceutical drugs and be HIV+.

#### Correlates of HCV infection:

In univariate analysis, HCV prevalence was significantly higher among those who were not married, had a history of a tattoo, history of injection at a dealer’s place, and had a longer duration of injecting drugs (Table II). HCV antibody positive individuals also tended to be older than those who were HCV negative, but this difference was not significant. Frequency of alcohol consumption was negatively associated with HCV prevalence.

**Table II:** Unadjusted and adjusted prevalence ratios of HCV antibody positivity (n=1158)^a^

	Unadjusted PR	95% CI	Adjusted PR	95% CI
Age (per 10 years)	1.08	1.00 - 1.15	0.93	0.85 - 1.02
Marital status				
Married/living with partner	1	-	1	-
Single	1.25	1.12 - 1.39	1.21	1.09 - 1.35
Separated	1.58	1.32 - 1.88	1.22	1.04 - 1.42
Divorced/widowed	1.26	0.86 - 1.86	1.19	0.84 - 1.67
Educational attainment				
None	1	-	1	-
Primary	1.02	0.88 - 1.17	1.05	0.92 - 1.20
Secondary	1.12	0.97 - 1.30	1.06	0.92 - 1.22
High school or more	1.24	1.05 - 1.46	1.23	1.05 - 1.45
Frequency of alcohol use				
Never	1	-	1	-
< 1 day/wk	0.94	0.83 - 1.06	0.93	0.83 - 1.04
1-2 days/wk	0.76	0.65 - 0.90	0.82	0.71 - 0.95
>3 days/wk	0.59	0.51 - 0.70	0.64	0.54 - 0.75
History of tattoo				
No	1	-	1	-
Yes	1.41	1.25 - 1.60	1.26	1.13 - 1.44
History of injection at dealer’s place				
No	1	-	1	-
Yes	1.50	1.35 - 1.67	1.26	1.14 - 1.41
Years of injection drug use				
≤ 1	1	-	1	-
1-5	1.84	1.09 - 3.10	2.67	1.40 - 5.09
6-10	3.89	2.35 - 6.43	4.93	2.62 - 9.27
>10	4.31	2.61 - 7.10	5.82	3.09 - 10.98

a Prevalence ratios estimated using Poisson regression with robust variance estimation; Adjusted for age, marital status, education, alcohol frequency, history of tattoo and injection at dealer’s, and years of injection drug use. HCV, hepatitis C virus; PR, prevalence ratio; CI, confidence interval

In multivariate analysis HCV prevalence increased significantly with increasing duration of injection. Compared to those who injected for <1 yr, HCV prevalence was significantly higher in those who injected for 1-5 yr (PR: 2.67; 95% CI: 1.40, 5.09), 6-10 yr (PR: 4.93; 95% CI: 2.62, 9.27), and >10 yr (PR: 5.82; 95% CI: 3.09, 10.98). History of tattoo (PR: 1.26; 95% CI: 1.13, 1.44) and having ever injected at a dealer’s place (PR: 1.26; 95% CI: 1.14, 1.41) were also positively associated with higher HCV prevalence. Increasing frequency of alcohol consumption was negatively associated with HCV prevalence. Compared to those who did not drink, those who reported drinking 1-2 days per week (PR: 0.82; 95% CI: 0.71, 0.95) and >3 days per week (PR: 0.64; 95% CI: 0.54, 0.75) had significantly lower HCV prevalence (Table II).

#### HCV virology:

Of the 400 HCV antibody positive IDUs, 281 (70.3%) were persistently infected with HCV. The median HCV RNA level among those with detectable HCV RNA was 1.24 million IU/ml (IQR: 0.42 - 2.67); no factors were significantly associated with HCV RNA level except for chronic HBV infection (-0.57; 95% CI: -1.09, -0.05).

In univariate analysis, HBV infection and marijuana use were protective against persistent HCV infection (Table III). Neither age, HIV infection, alcohol frequency, nor recent drug use were significantly associated with HCV persistence. In multivariate analysis HBV co-infection (PR: 0.38; 95% CI: 0.23, 0.64) and marijuana use remained protective (PR 0.83 for < daily use vs. no use; 95% CI: 0.71, 0.97).

**Table III:** Unadjusted and adjusted prevalence ratios of HCV viral persistence (n=400)^a^

Variable	Unadjusted PR	95% CI	Adjusted PR	95% CI
Age (per 10 years)	1.06	0.95 - 1.17	1.05	0.94 – 1.18
HIV status				
Negative	1	-	1	-
Positive, CD4>350 cells/μ	0.88	0.75 - 1.03	0.86	0.74 – 1.0
Positive, CD4≤350 cells/μ	0.93	0.75 - 1.13	0.90	0.73 – 1.11
Chronic HBV infection				
No	1	-	1	
Yes	0.38	0.23 - 0.65	0.38	0.23 – 0.64
Years of injection drug use				
≤ 1 yr	1	-	1	-
1-5 yr	1.52	0.75 - 3.07	1.55	0.77 – 3.08
6-10 yr	1.31	0.65 - 2.66	1.41	0.71 – 2.80
>10 yr	1.44	0.72 - 2.90	1.49	0.75 – 2.96
Alcohol frequency				
Never	1	-		
< 1 day/wk	0.99	0.85 - 1.16		
1-2 days/wk	0.97	0.79 - 1.18		
< 3 days/wk	0.94	0.77 - 1.15		
Recent marijuana use				
No	1	-	1	-
<daily	10.83	0.71 - 0.98	0.83	0.71–0.97
≥daily	0.89	0.77 - 1.03	0.923	0.80–1.06
Recent injection drug use				
None	1	-		
< daily	1.03	0.87 - 1.22		
≥ daily	0.93	0.76 - 1.14		

a Prevalence ratios estimated using Poisson regression with robust variance estimation; Adjusted for HIV status, HBV status, alcohol frequency, age, marijuana use in the prior one month and injection drug use in the prior one month. HCV, hepatitis C virus; PR, prevalence ratio; 95% CI, 95% confidence interval; HIV, human immunodeficiency virus; HBV, hepatitis B virus

Among the 13 samples that were sequenced, 11 (85%) were infected with HCV genotype 3a and 2 (15%) genotype 1b.

#### Prevalence of liver disease:

**Fig. 2:**
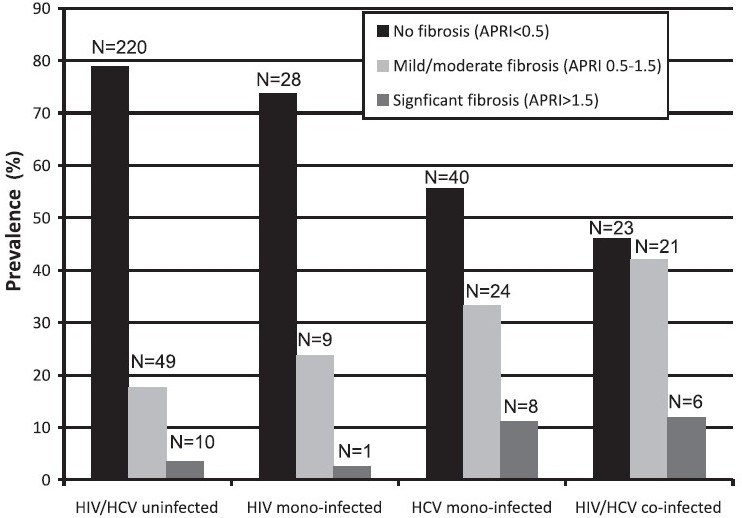
Prevalence of significant fibrosis by HIV and chronic hepatitis C virus infection (n=927). Chronic HCV infection was defined by the presence of detectable HCV RNA; HCV uninfected individuals included those who were HCV antibody negative and those who were HCV antibody positive and had undetectable HCV RNA. 231 individuals who were HCV antibody positive but did not have HCV RNA testing were excluded. The presence of liver fibrosis was estimated using the AST to platelet ratio index (APRI).

The 557 participants who returned at 30 months were not different from those who did not return with respect to age, general demographics, years of injection drug use, HIV and/or HCV antibody status. According to validated cut-offs, 372 had no disease, 146 had mild/moderate fibrosis and 39 had significant fibrosis. The prevalence of significant liver disease varied by HCV RNA and HIV antibody status at baseline: [HIV/HCV uninfected: 10 (4%); HCV mono-infected (HCV RNA+): 8 [(11%); HIV antibody positive: 1 (3%); HCV RNA+/HIV antibody positive: 6 (12%)]. A total of 118 (21%) had unknown HCV RNA status (Fig. 2).

Characteristics of persons with significant fibrosis (n=39) were further examined compared with those with no or mild/moderate liver disease (n=518). Detectable HCV RNA at baseline, HIV infection, duration of injection drug use, proportion of visits where alcohol was consumed ≥ 3 days per week and per cent of visits where other pharmaceutical injection drug use (non-buprenorphine) was reported, were all associated with higher prevalence of significant fibrosis. Marijuana use was associated with lower prevalence of fibrosis (Table IV).

**Table IV:** Unadjusted and adjusted prevalence ratios of significant fibrosis (n=557)^a^

	Unadjusted PR	95% CI	Adjusted PR*	95% CI
Age (per 10 years)	1.25	-	1.43	0.68 - 1.58
Years of injection drug use				0.97 - 1.08
≤ 1	1	-	1	-
1-5	1.75	0.22 - 13.8	1.41	0.18 - 10.8
6-10	2.29	0.30 - 17.6	1.38	0.28 - 16.9
>10	4.11	0.57 - 29.6	2.18	0.28 - 16.9
HIV/HCV				
HIV/HCV uninfected	1		1	
HIV mono-infected	0.73	0.10 - 5.59	1.17	0.15 - 8.96
HCV mono-infected	3.10	1.27 - 7.58	3.34	1.23 - 9.07
HIV/HCV co-infected	3.35	1.27 - 8.81	3.92	1.29 - 11.9
Chronic HBV infection	1.50	0.69 - 3.27	1.49	0.69 - 3.22
% visits heavy alcohol use reported	4.04	1.45 - 11.29	6.62	2.68 - 16.3
% visits daily marijuana use reported	0.42	0.19 - 0.94	0.37	0.16 - 0.83
% visits heroin injection	2.03	0.49 - 8.38		
% visits buprenorphine injection	0.87	0.05 - 14.3		
% visits non-buprenorphine pharmaceutical drug use	5.13	1.35 - 19.5	2.15	0.59 - 7.94

a Prevalence ratios estimated using Poisson regression with robust variance estimation; Adjusted for age, years of injection drug use, HIV/ HCV status, HBsAg status, % of visits where heavy alcohol use, marijuana use, and other non-buprenporphine pharmaceutical drug use was reported. % visits daily marijuana use reported was calculated by dividing the number of follow up of visits where daily marijuana use was reported, divided by the total number of follow up visits that each participant attended. % visits heavy alcohol use reported, % visits heroin injection, % visits buprenorphine injection and % visits other non-buprenorphine pharmaceutical drug use where calculated in a similar fashion. PR, prevalence ratio; 95% CI, 95% confidence interval; HIV, human immunodeficiency virus; HCV, hepatitis C virus; heavy alcohol use was defined as drinking alcohol at least 3 days a week

In multivariate analysis, prevalence of significant fibrosis was significantly higher in HCV mono-infected individuals (PR: 3.34; 95% CI 1.23 to 9.07) and HIV/HCV co-infected individuals (PR: 3.92; 95% CI 1.29 to 11.1) compared with HIV/HCV uninfected persons. Heavy alcohol use was also positively associated with significant fibrosis (PR for a one per cent increase in visits where heavy alcohol use was reported: 6.62; 95% CI: 2.68, 16.3). Significant fibrosis was negatively associated with daily marijuana use (PR for a one per cent increase in visits where daily marijuana use was reported: 0.37; 95% CI: 0.16, 0.83).

## Discussion

We observed a high burden of HCV among IDUs in south India. The low prevalence of significant liver disease in this cohort may reflect the relatively young age, short duration of injection and high rate of AIDS-related mortality^17^. However, disease progression is likely to increase as IDUs age and access to HAART improves, particularly given the high prevalence of cofactors for HCV progression including HIV and heavy alcohol use.

The burden of HCV infection seen in this study was consistent with data from India and other countries^1^. Further, correlates of HCV infection were similar to those reported elsewhere. Though HCV prevalence increased with duration of injection, the marked increases in the first two years of injection that have previously been observed^18^were not seen in the MIDACS, suggesting a window of opportunity for prevention of HCV acquisition.

In contrast to prior studies that identified associations between HCV viral persistence and age and HIV co-infection^19^, such associations were not observed in our study. There are multiple explanations for this discrepancy. First, the majority of IDUs acquire HCV prior to HIV; the impact of HIV on HCV viral persistence likely occurs only when a person becomes re-infected with HCV following repeated exposure^16^. Given the young age of this cohort, it is possible that not enough time has elapsed for persons to have acquired multiple HCV infections; an effect of HIV may manifest as this cohort ages. Further, the prevalence of daily injection was lower in this cohort than others suggesting less opportunity for repeated exposure^20^. Other possible explanations may include host or viral genetics.

Chronic HBV infection was associated with HCV viral clearance and with lower HCV RNA levels as has been seen previously^19^,^21^. The pathogenesis of HBV and HCV co-infection is incompletely understood; it is not clear whether HCV leads to increased clearance of HBV or vice-versa^21^.

Though prevalence of significant fibrosis was lower than what has been observed in other IDU cohorts, nearly 10 per cent had already progressed to significant liver disease. The burden of liver disease is likely to increase as this cohort ages, particularly given the high prevalence of HIV co-infection and heavy alcohol use^3^,^19^. Further, we have previously reported that after enrolling in the study and receiving voluntary counselling and testing, nearly 90 per cent stopped injecting^20^; however, a concurrent increase was observed in alcohol and marijuana use suggesting that users were substituting. The longer term consequences of this for liver disease could be severe.

Interestingly, negative associations were observed between marijuana use and liver disease which is in contrast to previous epidemiologic studies that have reported the opposite^22^. On one hand, two receptors on the surface of hepatocytes have been shown to be stimulated by marijuana but have opposite effects on liver fibrosis^23^,^24^. On the other hand, marijuana use may be a marker for some other behaviour that was incompletely measured. Further, given the nature of the data, fibrosis may have occurred years before reported use of marijuana and patterns of use may have changed over time.

Beyond substance use, a number of factors warrant concern for future liver disease progression. Currently, a few IDUs have received HAART, but this will change as access to HAART expands for IDUs in India. A typical first-line regimen in India and the developing world includes nevirapine (NVP), lamivudine (3TC) and stavudine (d4T). NVP has been associated with increased risk of hepatotoxicity among persons co-infected with HCV or HBV, and d4T has been associated with steatosis^25^. We have previously reported high rates of tuberculosis among HIV+ IDUs in this cohort^11^. Anti-tuberculosis regimens in India include rifampin which is also hepatotoxic^26^. IDUs in India also encounter other pathogens that are not prevalent in the developed world including malaria, leptospirosis, dengue and chikungunya, all of which have been associated with alterations in liver function^27^,^28^. Finally, malnutrition, which has also been associated with progression to cirrhosis^29^, is rampant; the median body weight among HIV+ IDUs was less than 50 kg^11^. These factors collectively suggest that the burden of liver disease is likely to increase in this population.

The predominant HCV genotype was 3a. Currently, the availability of treatment for HCV in India is limited. However, when treatment becomes accessible, it is encouraging that the predominant genotype is one that has been highly responsive to current treatment regimens and may require only 28 wk (vs. 52)^30^. Further sampling will be needed to determine whether subtype 3a is truly prevalent in all regions; this convenient yet statistically valid^13^ sample represents IDUs only in Chennai.

The limitation of this study was the cross-sectional data for HCV prevalence and persistence which prevented establishment of temporality of exposures and outcomes. Even in the liver fibrosis analysis where we used longitudinal exposure data, it is possible that the occurrence of fibrosis preceded predictors. Further, the majority of data relied on participant self-report which may be subject to information bias. The current gold standard for assessing liver fibrosis remains the liver biopsy which could not be done in this study. While APRI and other non-invasive markers have been validated in developed countries, APRI has not been validated in India.

In conclusion, a high burden of HCV, predominantly subtype 3a was observed in this cohort of IDUs in southern India. Some differences in terms of HIV/HCV interactions were observed in this cohort warranting further investigation. Though most IDUs did not progress to significant liver disease, a number of factors including alcohol, marijuana and use of hepatotoxic HIV and TB medications place this population at high risk for progression of liver disease. Strategies to address issues of HCV co-infection among IDUs need to be included in all HIV and TB treatment programmes targeted at IDUs.
